# Flaviviruses in Game Birds, Southern Spain, 2011–2012

**DOI:** 10.3201/eid1906.130122

**Published:** 2013-06

**Authors:** Francisco Llorente, Elisa Pérez-Ramírez, Jovita Fernández-Pinero, Ramón Soriguer, Jordi Figuerola, Miguel Ángel Jiménez-Clavero

**Affiliations:** Instituto Nacional de Investigación y Tecnología Agraria y Alimentaria, Valdeolmos, Spain (F. Llorente, E. Pérez-Ramírez, J. Fernández-Pinero, M.Á. Jiménez-Clavero);; Consejo Superior de Investigaciones Científicas, Seville, Spain (R. Soriguer, J. Figuerola)

**Keywords:** Bagaza virus, flaviviruses, game bird, partridge, pheasant, Spain, viruses, Usutu virus, West Nile virus

**To the Editor:** Certain arthropod-borne epornitic flaviviruses, namely, West Nile virus (WNV) and Usutu virus (USUV), have spread recently in parts of Europe (*1*,*2*). In southern Spain, the emergence of a third virus of this type, known as Bagaza virus (BAGV), is of concern (*3*). Because of the outbreaks in 2010 in Cádiz (southern Spain) of WNV infection, which affected birds, horses, and humans, and of BAGV infection, which affected game birds (partridges and pheasants), and the reported presence of USUV in mosquitoes in this area (*4*), a surveillance program was implemented in partridges and pheasants during the next hunting season (October 2011–February 2012) to assess the possible circulation of these 3 flaviviruses in the area.

Serum samples and brain tissue from 159 hunted-harvested wild red-legged partridges (*Alectoris rufa*) and 13 common pheasants (*Phasianus colchicus*) were collected on 12 hunting properties from Cádiz (Technical Appendix Figure). All sampled birds were reared and shot in the wild. The age of the partridges was determined according to plumage characteristics.

Presence of antibodies against WNV was tested with a commercial epitope-blocking ELISA (Ingezym West Nile Compac, INGENASA, Madrid, Spain) (*5*). Virus-neutralization titers against WNV (strain Eg-101), BAGV (strain Spain/2010), and USUV (strain SAAR1776) were determined by micro virus neutralization test (VNT) as described (*6*).

Viral genome in brain tissue samples was examined by heminested pan-flaviviral reverse transcription PCR (*7*). All 172 tissue homogenates examined were negative by this test.

Overall seroprevalence for WNV by epitope-blocking ELISA was 29%. Prevalence of neutralizing antibodies measured by VNT was 23% for WNV, 15% for BAGV, and 10% for USUV. Seroprevalence rates were higher for pheasants than for partridges for WNV (Fisher exact test, p = 0.0003), BAGV (p<0.0001), and USUV (p<0.0001) (Table). The significance of this result is uncertain, given that just 2 hunting areas were sampled for pheasants.

**Table:** Results of serologic studies in red-legged partridges and common pheasants, southern Spain, 2011–2012*

Species, no.	Age	ELISA, WNV	VNT titers†			Interpretation‡
			WNV	BAGV	USUV	
Partridges (*Alectoris rufa*), n = 159						
6	Juvenile	+	**10–20**	<5	<5	WNV
7	Adult	+	**10–20**	<5	<5	WNV
1	Adult	+	**160**	**20**	**20**	WNV
4	Not determined	+	**10–80**	<5	<5	WNV
1	Not determined	+	**80**	<5	**10**	WNV
2	Juvenile	+	**10**	**40**	<5	BAGV
1	Juvenile	+	**20**	**80**	**10**	BAGV
1	Juvenile	–	<5	**160**	**10**	BAGV
2	Adult	+	**20, 40**	**80, 320**	**10**	BAGV
1	Adult	+	<5	**20**	<5	BAGV
1	Adult	+	<10‡	**40**	<10‡	BAGV
1	Adult	–	<5	**40**	<5	BAGV
1	Adult	+	<5	<5	**40**	USUV
2	Juvenile	+	**10, 20**	**20, 40**	<5	Flavivirus
3	Adult	+	<5	<5	<5	Flavivirus
2	Adult	+	**20, 80**	**80, 160**	**40**	Flavivirus
1	Adult	+	**20**	<5	**10**	Flavivirus
1	Adult	+	**160**	<20‡	**80**	Flavivirus
1	Adult	+	**40**	**40**	<10‡	Flavivirus
1	Adult	–	<10‡	**20**	<10‡	Flavivirus
3	Not determined	+	<5	<5	<5	Flavivirus
1	Not determined	+	<5	**10**	<5	Flavivirus
Pheasants (*Phasianus colchicus*), n = 13						
1	Adult	+	**80**	**20**	**10**	WNV
2	Not determined	+	**10, 20**	<5	<5	WNV
2	Adult	+	**10, 80**	**80, 640**	**20**	BAGV
1	Adult	–	<5	**20**	<5	BAGV
3	Adult	+	**20–40**	**20–160**	**40–160**	Flavivirus
1	Adult	+	**10**	<5	<5	Flavivirus
1	Adult	–	<5	**10**	<5	Flavivirus
1	Adult	Not determined	<20‡	<20‡	**40**	Flavivirus
Positive, no. (%)						
Partridges		40 (25)	31 (19)	17 (11)	11 (7)	
Pheasants		9 (69)	9 (69)	8 (62)	7 (54)	
Total		49 (28)	40 (23)	25 (15)	18 (10)	

*WNV, West Nile virus; VNT, virus neutralization test; BAGV, Bagaza virus; USUV, Usutu virus; +, positive; –, negative. **Boldface** indicates VNT-positive serum. †Serum samples were titrated from 5 to 1,280 dilutions and neutralization titers of >10 were considered positive. ‡Differentiation was based on comparison of VNT titers obtained in parallel against the 3 flaviviruses: the neutralizing immune response observed was considered specific when VNT titer for a given virus was >4-fold higher than titers obtained for the other viruses; samples showing VNT titer differences <4-fold between the viruses examined were considered positive for flavivirus but not conclusive for any specific virus. ‡VNT at the indicated (or lower) dilution(s) could not be determined because of cytotoxic effect caused by the sample.

Neutralizing antibodies to >1 flavivirus were detected in 15 of the 45 VNT-positive partridges and in 6 of the 12 VNT-positive pheasants (Table). Specificity, as determined by neutralizing antibodies titer comparisons (*8*), showed virus-specific neutralizing antibodies to WNV, BAGV, and USUV in 19 partridges, 9 partridges, and 1 partridge, respectively, in 3 pheasants to WNV and in another 3 pheasants to BAGV (0 to USUV). Serum from 9 partridges and 6 pheasants remained inconclusive (neutralizing antibodies titer differences <4-fold [*8*]). WNV-reacting antibodies by ELISA were shown in 11 of 12 hunting properties (Technical Appendix Figure). In all locations but 1, ELISA-positive results were confirmed by VNT for NT-Abs to WNV, BAGV, or USUV. Of them, neutralizing antibodies to only WNV were detected in 2 locations, whereas neutralizing antibodies to at least 2 (WNV/USUV or WNV/BAGV) of the 3 flaviviruses were detected in 8 locations. Within these locations, flavivirus-specific NT-Ab responses were differentiated in several samples: neutralizing antibodies to either WNV or BAGV were detected in samples from 6 locations, whereas samples with neutralizing antibodies to either WNV or USUV were detected in 1 location.

Analysis of VNT results in juvenile partridges showed specific neutralizing antibodies to WNV (13%) or BAGV (9%); 4% of these samples were positive for flavivirus but inconclusive for any of the flaviviruses tested (Table). Overall, these results indicated recent circulation of 3 different epornitic flaviviruses—WNV, USUV, and BAGV—in resident game birds in Cádiz, the southernmost province in Spain. A high proportion of birds showed neutralizing antibodies to >1 flavivirus. Some are likely to be attributable to cross-neutralization, although co-infection cannot be ruled out because the results showed co-circulation of >1 flavivirus in game birds in most locations studied. Furthermore, the presence of specific neutralizing antibodies in juvenile partridges indicated that WNV and BAGV circulated in the area 1 year after the outbreaks of 2010. For USUV, specific neutralizing antibodies were detected only in an adult partridge, indicating infection acquired during the previous years. Nevertheless, this finding does not rule out recent co-circulation of USUV together with the other 2 viruses in the same game bird populations, considering that USUV has been repeatedly detected in mosquitoes in nearby areas (*4*).

Evidence of infection by at least WNV and BAGV in 2 consecutive seasons strongly supports the premise that these viruses overwintered in the area. Capability of WNV to overwinter in southern Europe was shown in Italy during 2008–2009 (*9*) and in Spain during 2007–2008 (*10*). Overwintering of BAGV after its introduction into Spain could indicate a risk for its expansion in areas with similar climates (Mediterranean basin). The risk for dissemination of WNV, BAGV, or USUV infections not only to game birds, but also to other wildlife, domestic animals, and humans, calls for improvements in surveillance programs, particularly those that monitor susceptible hosts, such as game birds.

Technical Appendix Study area in Spain (province of Cádiz) showing the position of hunting properties analyzed.
