# Supplementary material for: Flaviviruses in Game Birds, Southern Spain, 2011–2012
Source: Emerg Infect Dis. 2013 Jun;19(6):1023–5. doi: 10.3201/eid1906.130122 (PMC3713840; doi:10.3201/eid1906.130122)
Supplement: Technical Appendix — Study area in Spain (province of Cádiz) showing the position of hunting properties analyzed. [file 13-0122-Techapp-s1.pdf]

Article DOI: http://dx.doi.org/10.3201/eid1906.130122

# Flaviviruses in Game Birds, Southern Spain, 2011–2012

## Technical Appendix

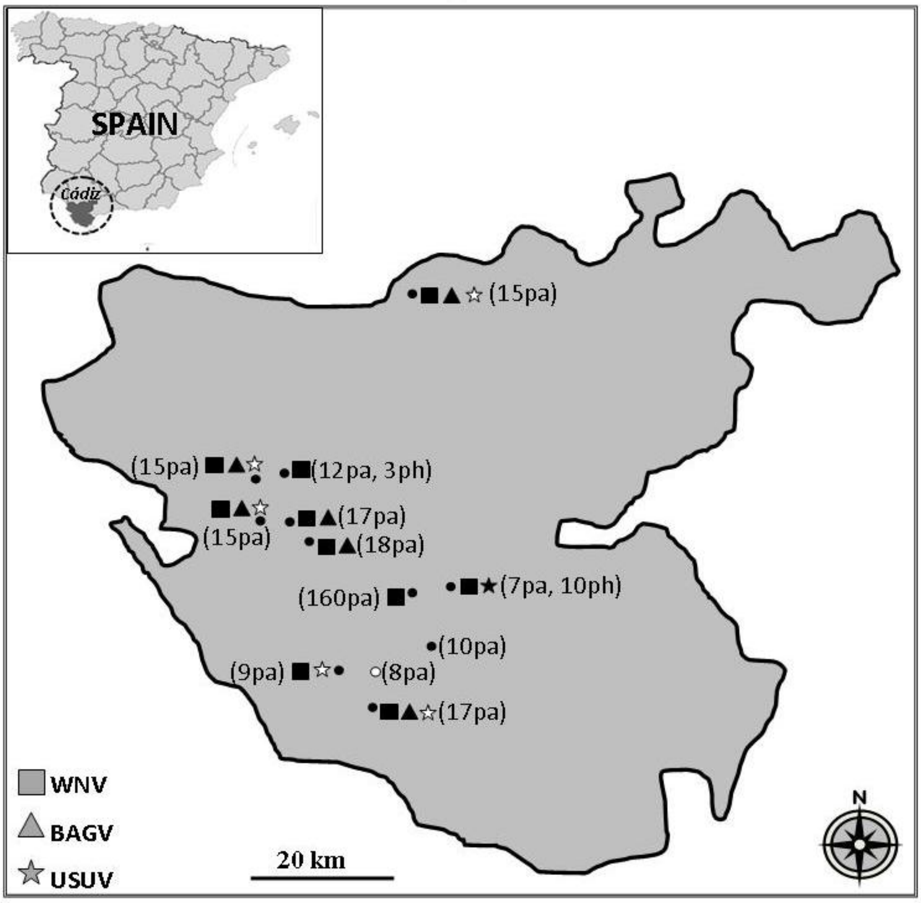


Technical Appendix Figure. Study area in Spain (province of Cádiz) showing the position of hunting properties analyzed (circles) with positive (closed circles) or negative (open circles) results to blocking ELISA. Presence of neutralizing antibodies to West Nile virus (squares), Bagaza virus (triangles) and Usutu virus (stars) is indicated. Open and closed symbols represent nonspecific (i.e., virus neutralization test titer difference <4-fold) and specific (≥4-fold) neutralizing antibody responses, respectively. The number of partridges (pa) or pheasants (ph) analyzed in each location is indicated between parentheses.
